# An Adenovirus-Vectored Influenza Vaccine Induces Durable Cross-Protective Hemagglutinin Stalk Antibody Responses in Mice

**DOI:** 10.3390/v9080234

**Published:** 2017-08-21

**Authors:** Eun Hye Kim, Gye-Yeong Han, Huan Nguyen

**Affiliations:** Viral Immunology Laboratory, International Vaccine Institute, SNU Research Park, 1-Gwanak-ro, Gwanak-gu, Seoul 08826, Korea; gyhan@ivi.int (G.-Y.H.); hnguyen@ivi.int (H.N.)

**Keywords:** influenza virus, vectored vaccine, cross-protection, stalk immunity, antibody, cytokine

## Abstract

Currently licensed vaccines against the influenza A virus (IAV) need to be updated annually to match the constantly evolving antigenicity of the influenza virus glycoproteins, hemagglutinin (HA), and neuramidiase (NA). Attempts to develop universal vaccines that provide broad protection have resulted in some success. Herein, we have shown that a replication-deficient adenovirus expressing H5/M2e induced significant humoral immunity against the conserved HA stalk. Compared to the humoral responses induced by an inactivated influenza vaccine, the humoral responses induced by the adenovirus-vectored vaccine against the conserved stalk domain mediated cross-protection against heterosubtypic influenza viruses. Importantly, virus inactivation by formaldehyde significantly reduced the binding of monoclonal antibodies (mAbs) to the conserved nucleoprotein (NP), M2e, and HA stalk. These results suggest that inactivation by formaldehyde significantly alters the antigenicity of the HA stalk, and suggest that the conformation of the intact HA stalk provided by vector-based vaccines is important for induction of HA stalk-binding Abs. Our study provides insight into the mechanism by which a vector-based vaccine induces broad protection by stimulation of cross-protective Abs targeting conserved domains of viral proteins. The findings support further strategies to develop a vectored vaccine as a universal influenza vaccine for the control of influenza epidemics and unpredicted pandemics.

## 1. Introduction

Currently available influenza A virus (IAV) vaccines require frequent changes in viral strain composition to address the continuous antigenic evolution of seasonal influenza viruses. However, these vaccines often provide poor immunity and are often not effective at preventing severe illness. The successes of these licensed vaccines to prevent influenza disease are limited by several factors including poor vaccine coverage, constant antigenic drift, and vaccine mismatch. Despite limitations on the data from retrospective clinical studies and a limited number of human challenge infection studies, variable protection against heterologous challenge after administration of inactivated influenza vaccine has been reported for a pediatric patient cohort [1]. In experimental settings, single or prime/boost vaccinations with inactivated vaccine did not induce a robust cross-reactive immune response, nor did they provide protection against heterologous influenza virus challenge in either mice or ferrets [2,3,4]. While a trivalent inactivated vaccine (TIV) fails to provide broad protection [3,5], live attenuated influenza vaccines (LAIV) have been shown to induce cross-protection. A robust immune response and protection conferred by LAIV was attributed to serum and mucosal antibody responses and cell-mediated immunity in ferrets [5,6,7,8]. These results also support the findings that LAIV is immunologically superior in children [3,9,10,11].

Replication-defective recombinant adenoviruses (rAd) have been developed as vectors for gene therapy or vaccines against human immunodeficiency virus (HIV), hepatitis virus, influenza virus, and certain types of cancers [8,12,13,14,15,16,17,18]. Adenovirus-vectored influenza vaccines have been reported to be safe [19,20,21], and have also been demonstrated to confer broad protection against multiple influenza virus subtypes [22,23,24,25,26,27].

Matrix protein 2 ectodomain (M2e) has been reported as an attractive, promising component of a broadly protective, universal influenza A vaccine [28]. The mechanisms of cross-protection by M2e-based vaccines [29] include induction of M2e-specific memory CD4 T cells that are broadly protective [30].

In this study, we have generated replication-defective human Ad serotype 5-derived vector encoding humanized full-length H5 HA, four tandem copies of the ectodomain (M2e) of the M2e (rAdH5/M2e) as a universal influenza A virus vaccine candidate. 

Attachment of influenza virus to sialic acids on cellular receptors and fusion of the viral and cell membranes are functions mediated by the HA globular head (HA1) and conserved stalk (HA2) domains, respectively [31]. Vaccines that target the conserved stalk domain for induction of cross-protection are supported by the encouraging results from animal models [27,32,33,34].

The cytokines interferon (IFN)-γ, interleukin (IL)-2, and IL-12 define a T helper 1 (Th1) response that stimulates production of serum immunoglobulin (Ig) G2A and IgG2B Ab in mice [34,35,36,37], whereas the cytokines IL-4, IL-5, IL-6, and IL-10 characterize a Th2 response [38] and stimulate secretion of IgG1, which is the main IgG Ab subclass produced by influenza-infected mice [34]. IL-6 induces inflammation and production of IL-4, which promotes differentiation of CD4+ T cells into effector Th2 cells and induction of B cell responses [17,39]. In the context of mouse vaccination model, decreasing IL-6 levels resulted in corresponding decreases in IL-4 and IL-6 levels, which had a significant impact on protective antibody responses [18]. IL-10 is known as a major immunomodulatory cytokine that can suppress Th1 cytokines, such as IL-2, and IFN-γ production, and impair T-cell responses [13,40]. These findings reveal that an ideal influenza vaccine should induce a desirable Th1/Th2 profile for induction of broadly protective humoral responses.

In this study, we examined the cross-protective immunity induced by immunization with the rAdH5/M2e vaccine. Herein, we show that immunization with an adenovirus-vectored influenza vaccine induced strong neutralizing Ab responses against the conserved hemagglutinin stalk domain and demonstrate that these vaccine-induced, Th2-type specific stalk-specific antibodies provide protection against influenza virus infection. Importantly, we provide evidence from studies that included vaccines treated with formaldehyde that the native conformation of the epitopes are necessary for induction of protective immunity. The results provide evidence of the advantages of using an adenovirus vector as a platform for the development of universal influenza vaccines and the generation of cross-protective antibodies for control of influenza.

## 2. Materials and Methods

### 2.1. Viruses

A/Aquatic bird/Korea/W81/2005 (H5N2), isolated from a wild bird in Korea 2006 and kindly provided by Young-Ki Choi of Chungbuk National University, Korea, was adapted by multiple passages (15 times) in Balb/c mice. After the final passage, a single plaque isolated by plaque purification on MDCK cells was amplified in embryonated chicken eggs. The mouse LD_50_ of the H5N2 virus was determined for the challenge experiment as described elsewhere [41]. The mouse-adapted A/PR/8/34 (PR8) (H1N1) virus was prepared from lung homogenates of intranasally infected mice prior to use for challenge infections.

### 2.2. Recombinant Adenoviral Vectored Vaccine

Influenza M2 and HA antigen sequences were derived from A/Vietnam/1230/2004 (H5N1) (GenBank AY651388 and AY651334, respectively). The recombinant adenovirus (rAd) vector encoding H5 HA was selected for immediate evaluation as a vaccine candidate against a potential H5N1 pandemic. The codon optimized H5 HA sequence (synthesized by GenScript, Piscataway, NJ, USA) was cloned into the pShuttle/CMV plasmid, which allowed for homologous recombination with a plasmid encoding an Ad backbone in BJ5183 *Escherichia coli* (*E. coli.*) rAd vectors were subsequently generated by transfecting recombinant plasmid containing the bioengineered Ad genomes encoding the transgenes into 293 cells. The vectors were mass-produced, purified and titrated according to the AdEasy manual. The genomes were sequenced to confirm the presence of M2e and HA genes and their flanking Ad sequences. M2e and HA of H5N1 origin were validated in lysates prepared from transduced HeLa cells by western blot analysis using Abs to M2 kindly provided by W. Gerhard, the Wistar Institute, Philadelphia, PA, USA and H5-specific IgY [42], respectively.

### 2.3. Animals

All animal experiments conformed to protocols approved by the International Vaccine Institute Institutional Animal Care and Use Committee (IACUC PN 2015-008, 15 July 2015). Female Balb/c mice aged 6–8 weeks were purchased from Orient Bio Inc. (Orient Inc., Gyeonggi-do, Korea). Animals were housed in pathogen-free barrier facilities with a 12-h dark and light cycle and free access to water and food.

### 2.4. Cell Lines

Madin–Darby canine kidney (MDCK) cells (ATCC #CCL-34, Manassas, VA, USA) were maintained in ATCC-formulated Eagle’s Minimum Essential Medium (ATCC, Manassas, VA, USA) containing 10% fetal bovine serum (Gibco, HyClone, Grand Island, NY, USA) and penicillin/streptomycin (Gibco, Life Technology, Grand Island, NY, USA; 100 units/mL and 100 µg/mL, respectively).

### 2.5. Protein, Recombinant Chimeric HA Protein and Mouse Monoclonal Antibodies (mAbs)

The M2e construct encoding three tandem copies of M2e conjugated to the C-terminal sequence of M2 protein of influenza A/Puerto Rico/8/34 (H1N1) virus was kindly provide by Manki Song, International Vaccine Institute, Korea [43]. The baculovirus-expressed chimeric HA cH9/1 protein contains the stalk domain of the H1 A/PR/8/34 (PR8) HA and the globular head domain of the H9 A/guinea fowl/Hong Kong/WF10/99. The mouse mAbs 7B2 specific for the HA globular head of A/California/4/09 (CAL/09) H1N1, 6F12 specific for the HA stalk of A/PR8 (H1N1), and PY102 specific for the HA globular head of A/PR8 (H1N1) were kindly provided by Peter Palese, Icahn School of Medicine at Mount Sinai (ISMMS), New York. The mAbs were previously described in detail [44,45].

### 2.6. Generation of Convalescent Sera for Vaccine Candidates

Mice were anesthetized by intraperitoneal injection of 0.1 mL of a ketamine/xylazine mixture (0.15 mg/kg and 0.03 mg/kg, respectively), and then either i.n. immunized with 1 × 10^7^ PFU in 50 μL of rAdH5/M2e or with a sublethal dose of live mouse-adapted A/PR/8/34 (PR8) (H1N1) or FluMist^®^ (MedImmune, Gaithersburg, MA, USA) that contains four vaccine virus strains: an A/H1N1 strain, an A/H3N2 strain and two B strains; or intramuscularly immunized (i.m.) with commercial trivalent inactivated vaccine (TIV, Vaxiflu, Dong-A Pharmaceutical, Seoul, Korea, Injection prefilled syringe) that contains purified inactivated influenza antigen type A (A/California/7/2009X-179A (H1N1)), (A/South Australia/55/2014IVR-175 (H3N2)), and purified inactivated influenza antigen B, diluted in 50 μL of PBS per mouse, respectively. Formaldehyde-inactivated PR8 virus (FiPR8 was prepared by treatment of egg-grown PR8 with 0.02% formaldehyde overnight followed by formaldehyde removal by dialysis. Where indicated, mice were intranasally immunized with 50 uL containing an equivalent of 2 × 10^7^ PFU of inactivated PR8 virus mixed with 2 µg of cholera toxin (FiPR8+CT) (List Biological Laboratories, Inc., Campbell, CA, USA). For all immunizations, mice were primed and boosted with the same procedure. For the long-term immunization, we used same dose of live PR8, and rAdH5/M2e, and mice received a booster immunization with FiPR8+CT, respectively. Four weeks after the last immunization, sera were collected and analyzed individually. For use in passive immunization experiments, sera collected from the same immunization group were pooled.

### 2.7. Vaccination/Challenge Experiments

Ketamine-anesthetized mice were intranasally inoculated with formaldehyde-inactivated PR8, live PR8, FluMist^®^, or rAdH5/M2e virus. The vaccine doses are specified in the figure legends. For lethal challenge infection, anesthetized mice were intranasally inoculated with 5LD_50_ of mouse-adapted A/PR/8/34 (PR8) (H1N1) or A/Aquatic bird/Korea/W81/2005 (H5N2) viruses.

### 2.8. Passive Immunization

The pooled sera samples were diluted 1:5 prior to intranasal immunization (i.n.) of anesthetized naïve mice. Six hours later, the recipient mice were challenged as described above.

### 2.9. Mouse Immunoglobulin Isotyping Magnetic Bead Panel

Isotyping of immunoglobulin in sera was performed using a magnetic bead panel 96-well plate assay (Millipore, Millipore Corporation, Billerica, MA, USA). Briefly, plates were treated with 25 µL of assay buffer at RT for 10 min. Then 50 µL of standard, control or undiluted serum samples were added to the appropriate wells. MILLIPLEX_MAP_ anti-mouse multi-immunoglobulin beads were then added to each well at RT for 15 min. Finally, 25 µL per well of diluted anti-mouse κ light chain PE was added at RT for 15 min. The results were obtained by reading the plates on a Luminex^®^ instrument (Luminex, Austin, TX, USA).

### 2.10. Enzyme Linked Immunosorbent Assay (ELISA)

Standard Enzyme Linked Immunosorbent Assay (ELISA) was performed for detection of antigen-specific Abs in sera. 96-Well MaxiSorp^TM^ Nunc Immuno plates (Nalgene Nunc International, Naperville, IL, USA) or Ni-NTA HisSorb plates (QIAGEN, GmbH, QIAGEN, Hilden, Germany) were coated with whole PR8 virus particles, M2e or chimeric HA protein (cH9/1) at a concentration of 2 µg/mL (100 μL per well). Plates were then treated with or without 0.2% of formaldehyde. Coated plates were blocked with PBS containing 0.1% Tween-20 (*v*/*v*) (TPBS) with 3% Bovine Serum Albumin (BSA). Each serum sample was pre-diluted 1:100 and serially diluted 1:5 in blocking buffer, and then adsorbed onto plates for 2 h. The bound immunoglobulins were detected with goat anti-mouse Ig (H+L) horseradish peroxidase-conjugated Abs, goat anti-mouse IgG, goat anti-mouse IgG1, goat anti-mouse IgG2A, goat anti-mouse IgG2B, and goat anti-mouse IgA (Southern Biotechnologies Associates, Inc., Birmingham, AL, USA) diluted 1:400 or 1:5000, respectively. As controls, the assays included mAbs 6F12, PY102, and 7B2 diluted to 2µg/mL and anti-M2 Ab diluted 1:1000. After the final wash step, tetramethylbenzidine (TMB) substrate was added and the reaction was stopped with an equal volume of 1M sulfuric acid. Absorbance was measured in a SPECTRAmax photometer (Molecular Devices, Sunnyvale, CA, USA) at 450 nm. Assay results were expressed as end-point titration values that were determined by the last dilution above the cutoff for the assay (Optical density (OD) 450 nm reaches plateau). To examine the effect of formaldehyde treatment on binding of epitope-specific mAbs, a modified ELISA was setup with influenza-virus-infected MDCK cells. Briefly, 1.5 × 10^4^ MDCK cells were added to each well of 96-well MaxiSorp Nunc Immuno plates (Nalgene Nunc International, Naperville, IL, USA) and incubated for 4 h at 37 °C, 5% CO_2_. The cells were then infected with 100 TCID_50_ of PR8 virus overnight. The plates were washed and fixed with 80% cold acetone in PBS and then treated with or without 0.2% formaldehyde. Binding of anti-NP or M2e-specific mAb (Millipore) was detected with goat anti-mouse IgG horseradish peroxidase (HRP)-conjugated Ab (BD) diluted 1:1000. The reactions were developed by addition of tetramethylbenzidine (TMB) substrate was and then stopped by addition of an equal volume of 1 M sulfuric acid. Absorbance was measured in a SPECTRAmax photometer at 450 nm. Secondary HRP Ab alone was used as a negative control.

### 2.11. Magnetic Luminex Screening Assay

Cytokines in sera were detected by the Mouse Magnetic Luminex Assay following the manufacturer’s instructions (R & D System, Inc., Minneapolis, MN, USA). The plates were developed by adding 50 µL of diluted streptavidin-PE to each well and incubating for 30 min at RT. The plates were read on a LUMINEX (Luminex, Austin, TX, USA).

### 2.12. Hemagglutination Inhibition (HI) Assay

Sera samples were treated with a receptor destroying enzyme (RDE-ΙΙ, Denka Seiken, Co., Ltd., Tokyo, Japan), which resulted in a final dilution of 1:10 before being tested in Hemagglutination inhibition (HI) assays. Two-fold serially diluted serum samples were incubated with an equal volume containing 8 HA units of the indicated virus in V shaped-bottom 96-well microtiter plates at 37 °C for 1 h. At the end of the incubation, freshly prepared 1% chicken red blood cells (CRBC) were added, and plates were mixed by agitation, covered, and allowed to set for 1 h at 4 °C. The HI titers were determined by the reciprocal of the last dilution, which contained non-agglutinated CRBC. Positive and negative control samples were included on each plate.

### 2.13. Microneutralization (MN) Assay

Neutralizing Ab titers were determined by microneutralization (MN) assays. Briefly, two-fold serially diluted serum samples were incubated with 100 TCID_50_ of viruses. The serum/antibody mixtures were then incubated at 37 °C for 1 h before adding to MDCK cells, and then the cultures were incubated at 37 °C for overnight. The presence of viral protein was detected by ELISA with anti-NP IgG Ab (Millipore). The neutralizing Ab titers were expressed as the reciprocal of the highest dilution of serum that gave 50% neutralization of 100 TCID_50_ of virus in MDCK cells.

### 2.14. Statistics

Statistical analyses were performed using Prism 5 (GraphPad, La Jolla, CA, USA). All values were plotted as averages with standard errors of the means. Student’s *t*-test, and ANOVA were used to determine the significant differences between two or multiple sets of experimental data, respectively. *p* Values of * < 0.01, ** < 0.005, and *** < 0.0001 were considered statistically significant.

## 3. Results

### 3.1. Influenza Vaccines Induce Neutralizing Th1/Th2 Vaccine-Specific Antibody Responses

**Figure 1:**
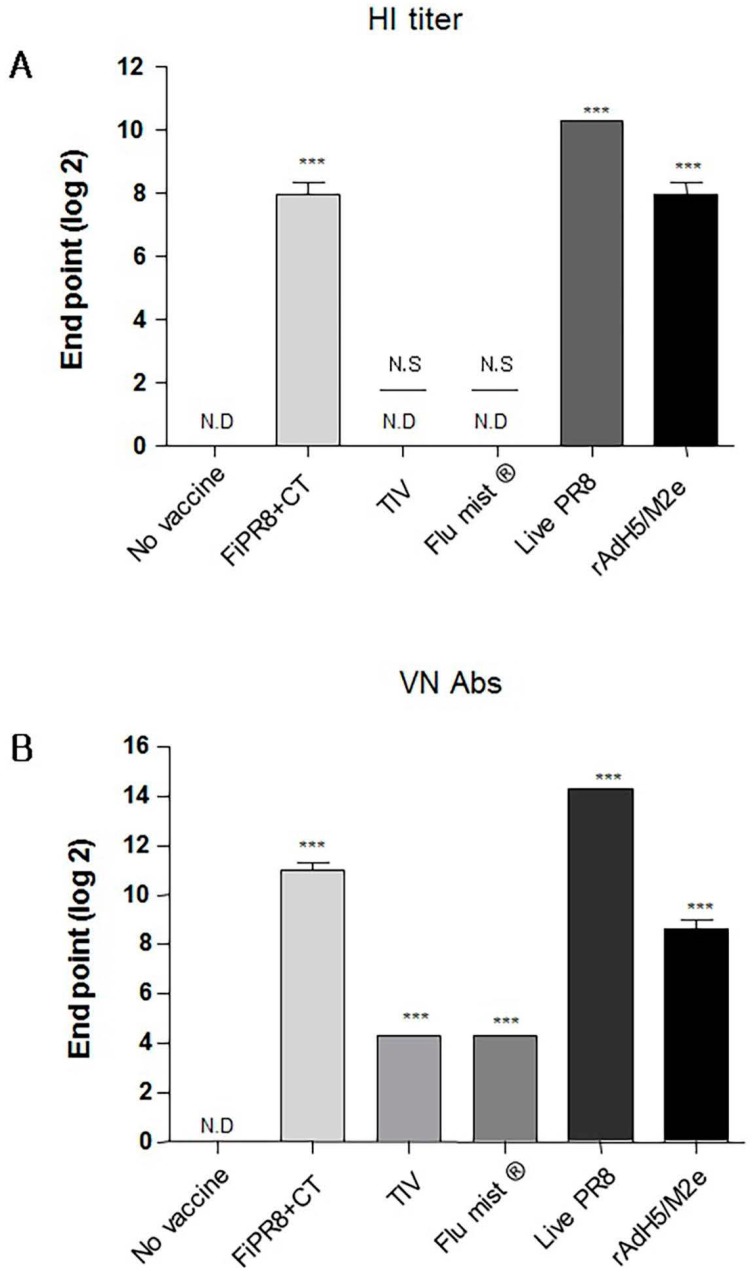

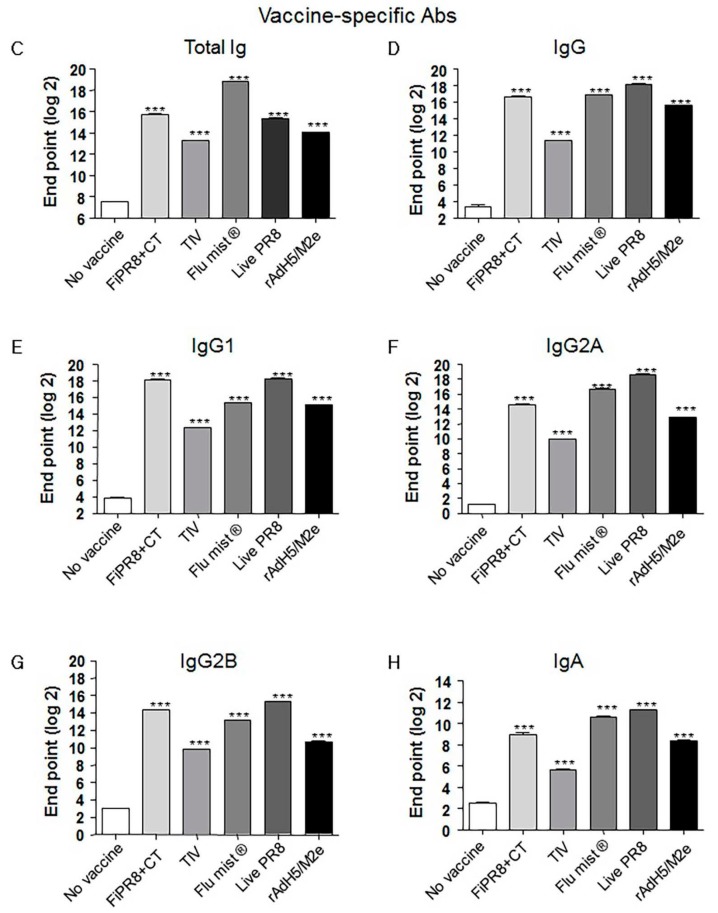
Adenovirus-vectored influenza vaccine induces a balanced T helper (Th)1/Th2 neutralizing antibody response. Balb/c mice were immunized with formaldehyde-inactivated PR8+CT (FiPR8+CT), live PR8 (H1N1), TIV, FluMist^®^ or, rAdH5/M2e. Four weeks post-immunization, sera were collected and the hemagglutination inhibiting (HI) titers (**A**), virus neutralization titers (**B**), and vaccine-specific total Ig, IgG, IgG1, IgG2A, IgG2B, and IgA antibodies (**C**–**H**) were determined. HI and VN titer of FiPR8+CT, TIV, Flu mist, and Live PR8 were tested against H1N1 and rAdH5/M2e was tested against H5N2. The values represent the mean ± SEM (vertical bars) of end point ELISA antibody titers determined from 5 mice per group (*** *p* < 0.0001).

To examine the antibody responses induced by our adenovirus-vectored influenza vaccine, mice were immunized with formalin-inactivated PR8 plus cholera toxin adjuvant (FiPR8+CT), live PR8, or adenovirus-vectored influenza vaccine, rAdH5/M2e. Groups of mice immunized with TIV (Trivalent Inactivated Vaccine) and FluMist^®^ were included to allow for comparison of our vectored vaccine with licensed vaccines. Following a single immunization, we determined the H1 or H5-specific serum antibody responses against A/PR/8/34 (PR8) (H1N1) or A/Aquatic bird/Korea/W81/2005 (H5N2) viruses, respectively, by hemagglutination inhibition assay and microneutralization assay. Immunization with FiPR8+CT, live PR8, or rAdH5/M2e or induced potent hemagglutination inhibiting (HI) and neutralizing antibody titers (Figure 1A,B, respectively). Immunization with TIV or FluMist^®^ failed to induce serum antibody responses. Considering that the balance of Th1/Th2 responses will impact the humoral responses induced by vaccines, we next examined the profiles of IgG and IgA antibody levels in the convalescent sera of mice immunized with these vaccines. We first analyzed the vaccine-specific total Ig and IgG-specific antibody responses for each immunization group by ELISA. As shown in Figure 1C,D, immunization with any of the indicated vaccinations stimulated elevated levels of vaccine-specific total Ig and IgG antibody responses. Interestingly, immunization with any of the indicated vaccines induced IgG subclass-specific responses without a notable bias of the Th1/Th2 balance (Figure 1E–G). In addition, all vaccinations induced corresponding vaccine-specific IgA antibody responses (Figure 1H).

### 3.2. Adenovirus-Vectored Vaccine Induce HA Stalk-Specific Antibodies

**Figure 2:**
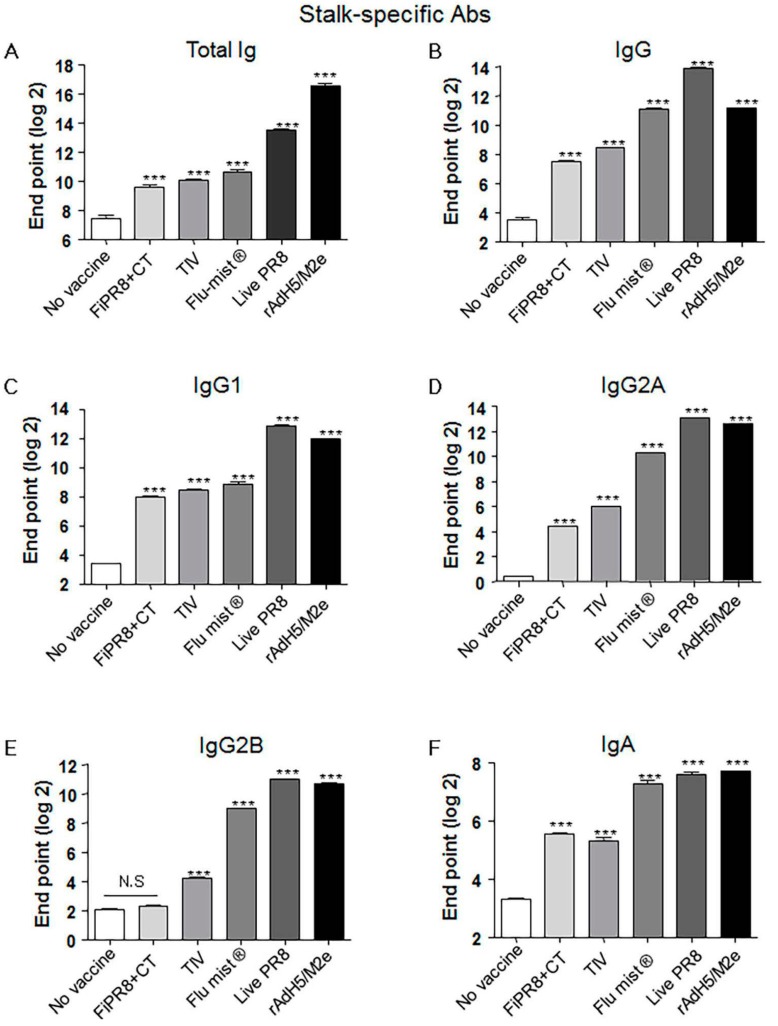
Adenovirus-vectored influenza vaccine induces a balanced Th1/Th2 antibody response against the HA stalk. Balb/c mice were intranasallyimmunized with formaldehyde-inactivated PR8+CT (FiPR8+CT), live PR8 (H1N1), TIV, FluMist^®^, rAdH5/M2e. The levels of serum hemagglutinin (HA) stalk-specific total immunoglobulin (Ig), IgG, IgG1, IgG2A, IgG2B, and IgA Abs were measured 28 days post-immunization by ELISA with baculovirus-expressed cH9/1 protein (**A**–**F**). The values represent the mean ± SEM (vertical bars) end point ELISA Ab titers determined from five mice per group (*** *p* < 0.0001).

Previous studies have demonstrated that monoclonal antibodies specific for the HA stalk domain or vaccination regimens that focus humoral responses on the HA stalk domain can provide confer cross-protection. Considering the importance of HA stalk-specific antibody responses to cross-protection, we next determined the levels of HA stalk-specific Abs in the mouse sera described in Figure 2 by ELISA, including recombinant baculovirus-expressed chimeric HA protein (cH9/1) containing the globular head of an H9 virus and the stalk domain of the H1 virus. As shown in Figure 2A,B, immunization of mice with rAdH5/M2e induced significant levels of HA stalk-specific total Ig or IgG Abs that were equal or superior to the antibody responses detected from the other vaccination groups. Interestingly, immunization with a live vaccine, i.e., FluMist^®^, live PR8, and rAdH5/M2e, generally induced the highest levels of stalk-specific IgG1, IgG2A, IgG2B, and IgA antibodies (Figure 2C–F).

### 3.3. Adenovirus-Vectored Influenza Vaccine Skews the Th1/Th2 Balance towards a Th2 Cytokine Response

**Figure 3:**
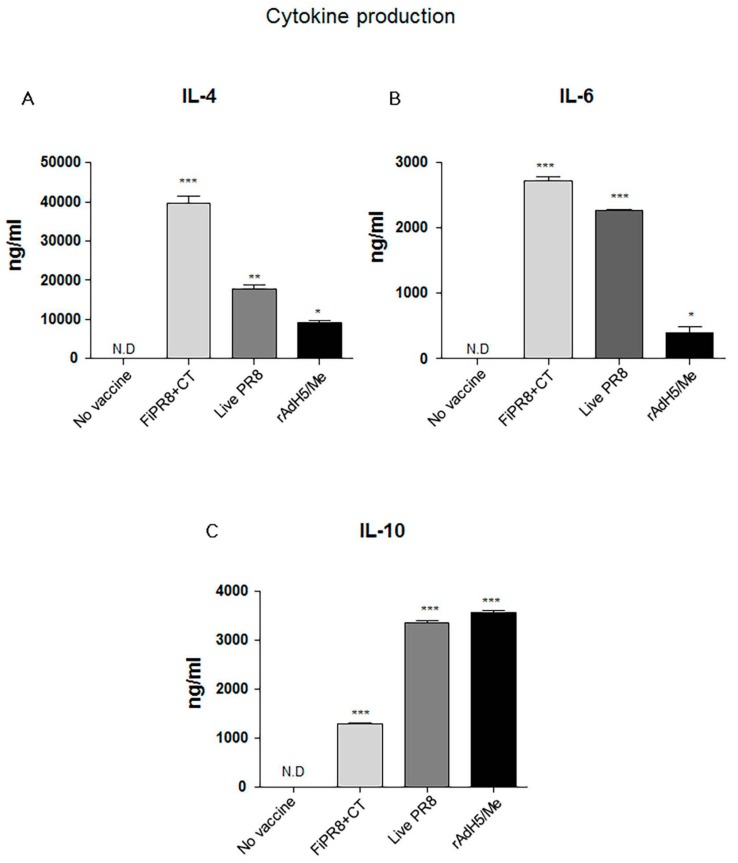
Adenovirus-vectored vaccine induces Th2 cytokine responses. Balb/c mice were intranasally immunized with formaldehyde-inactivated PR8+CT (FiPR8+CT), live PR8 virus, or AdH5/M2e. At four weeks post-immunization, sera were analyzed by Magnetic luminex screening assay (Millipore, Millipore Corporation, Billerica, MA, USA) to quantify levels of IL-4 (**A**), IL-6 (**B**), and IL-10 (**C**). The values represent the mean ± SEM (vertical bars) end point Ab titers determined from five mice per group. (* *p* < 0.01, ** *p* < 0.005, *** *p* < 0.0001).

The ELISA results suggested that immunization with the adenovirus-vectored influenza vaccine induced relatively balanced Th1 and Th2 immune responses as indicated by elevated levels of IgG2A and IgG2B, and IgG1 and IgA, respectively. Since the profile of cytokines can indicate the Th1/Th2 phenotype of the immune response, we examined the cytokine responses induced in the immunized mice to establish a correlation between the T helper response and antibody responses. IL-4 is key regulatory cytokine that induces differentiation of naive helper T cells into Th2 cells, stimulates differentiation of B cells into antibody secreting plasma cells, and promotes class switching from IgM to IgG1. Cholera toxin has been reported to strongly agonist of the IL-4 pathway and inducer of Th2 IgA antibody responses [46]. Not surprisingly, immunization with FiPR8+CT induced the highest IL-4 cytokine response of all vaccinations (Figure 3A). Since IL-6 is well known as a pro-inflammatory cytokine that can stimulate B cell proliferation [47], we analyzed IL-6 levels. As shown in Figure 3B, the adenovirus-vectored influenza vaccine, rAdH5/M2e, stimulated modest levels of IL-6 as compared to immunization with FiPR8+CT or live PR8. Since IL-10 is a major immune-modulatory cytokine that down regulates the expression of Th1 cytokines, and enhances B cell survival, proliferation, and antibody production [11,13], we also analyzed IL-10 levels. As shown in Figure 3C, immunization with rAdH5/M2e induced markedly higher levels of IL-10 that were comparable to levels induced by live PR8 or were higher than those induced by immunization with formaldehyde-inactivated PR8+CT. These cytokine data demonstrate that immunization with rAdH5/M2e induces Th2-type of cytokine responses that correlated with the observed IgG1 and IgA antibody titers, and balanced with Th1-type responses.

### 3.4. Adenovirus-Vectored Vaccine but Not Inactivated Virus Induce Cross-Protective Humoral Immunity

**Figure 4:**
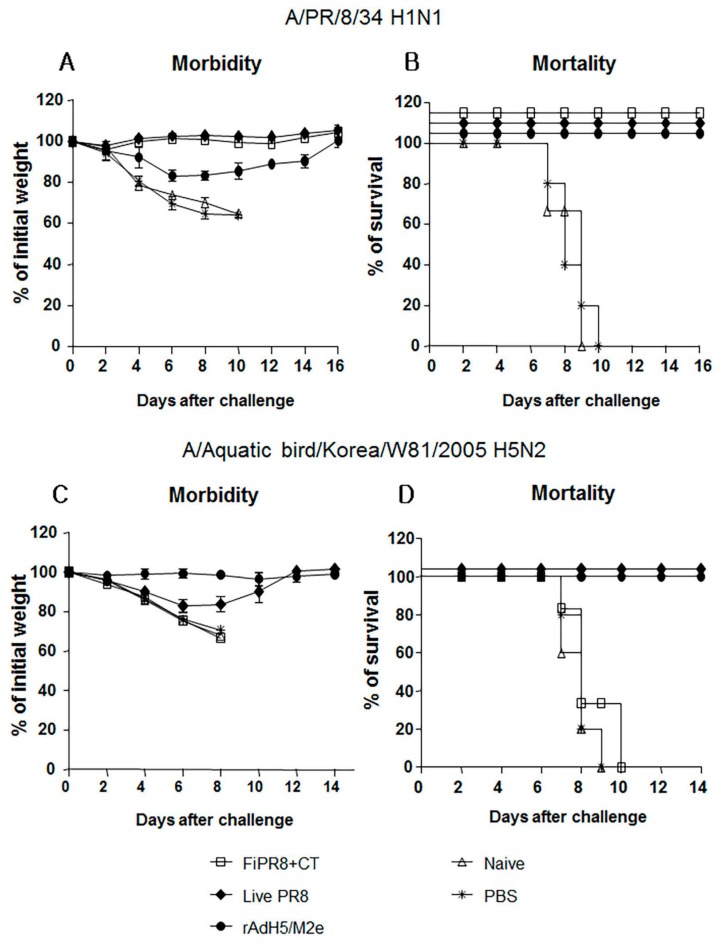
Passive immunization of immune sera from rAdH5/M2e vaccinated mice provided protection against influenza virus challenge infection. Anesthetized Balb/c mice were passively immunized by intranasal administration of sera from mice immunized with FiPR8+CT, live PR8 virus, or rAdH5/M2e vaccine. Control mice received sera from unvaccinated mice or phosphate buffered saline (PBS) only. Six hours later, recipient mice were infected with 5LD_50_ of mouse-adapted A/PR/8/34 (PR8) (H1N1) virus (**A**,**B**) or A/Aquatic bird/Korea/W81/2005 (H5N2) virus (**C**,**D**). Morbidity and mortality were monitored daily for two weeks after challenge infection. Body weights are expressed as the mean of the percent of starting body weight ± SD. Mortality is expressed as Kaplan-Meier survival curves. Each experimental group consisted of 5 mice per group. The data represent results from three independent experiments of five mice per group. The differences in body weight on days 5 and 6 between the groups immunized with live PR8 or vectored vaccine and formaldehyde-inactivated or unimmunized are statistically significant (*p* < 0.001).

After establishing that immunization of mice with rAdH5/M2e induces balanced Th1/Th2 antibody responses against the influenza virus hemagglutinin, we next examined the level of protection provided by this balanced humoral response against influenza virus challenge infection. To specifically focus our examination on the protective quality of the humoral immunity induced by our vaccination strategies, we passively immunized naïve mice with sera from mice immunized with FiPR8+CT, live PR8, or rAdH5/M2e. This passive immunization experiment eliminated any potential cytotoxic T cell responses that could have confounded our analysis of antibody-mediated protection from challenge infection. Following passive immunization of naïve mice, we then challenged the recipient mice by H1N1 or H5N2 challenge infection. As shown in Figure 4, immune sera from mice immunized with FiPR8+CT, live PR8, or rAdH5/M2e protected naïve recipient mice against challenge infection by an H1N1 virus (Figure 4A,B), whereas live PR8, or rAdH5/M2e protected naïve recipient mice against challenge infection by an H5N2 virus (Figure 4C,D). Sera transferred from animals immunized with FiPR8+CT protected naïve recipient mice from H1N1 infection, but not H5N2 infection. Importantly, passive immunization of mice with sera from rAdH5/M2e-immunized mice reduced morbidity and prevented mortality as a result of the heterosubtypic H1N1 virus infection. These results suggest that immunization with heterosubtypic H1N1 virus induced antibody-dependent cross-protection that was likely mediated by hemagglutinin-stalk specific antibodies.

### 3.5. rAdH5/M2e Vaccination Induced Durable Hemagglutinin Stalk-Specific Antibody Responses

**Figure 5:**
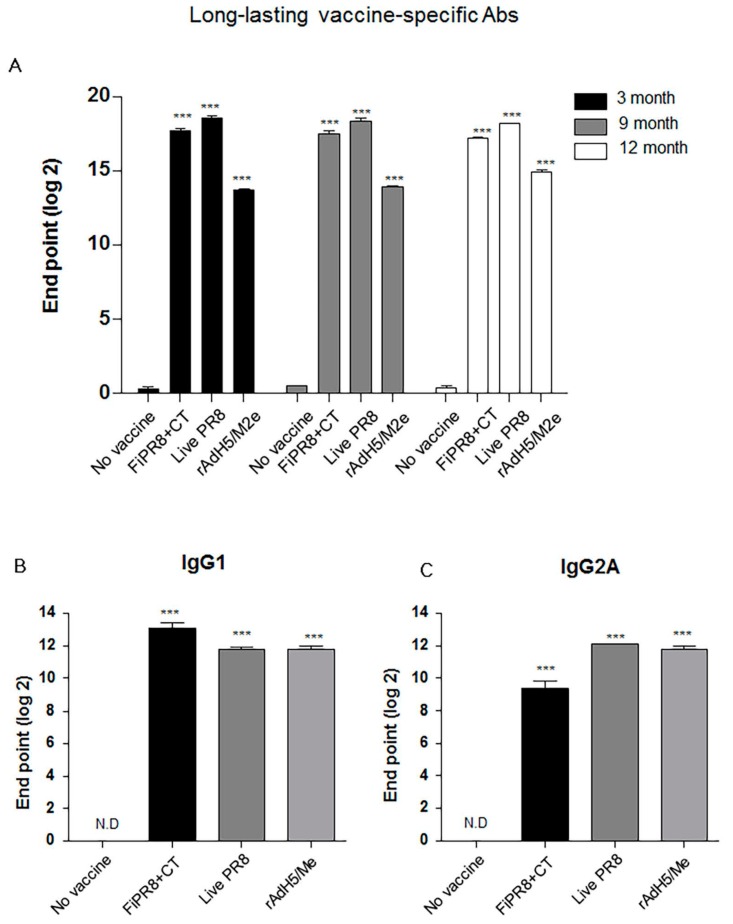

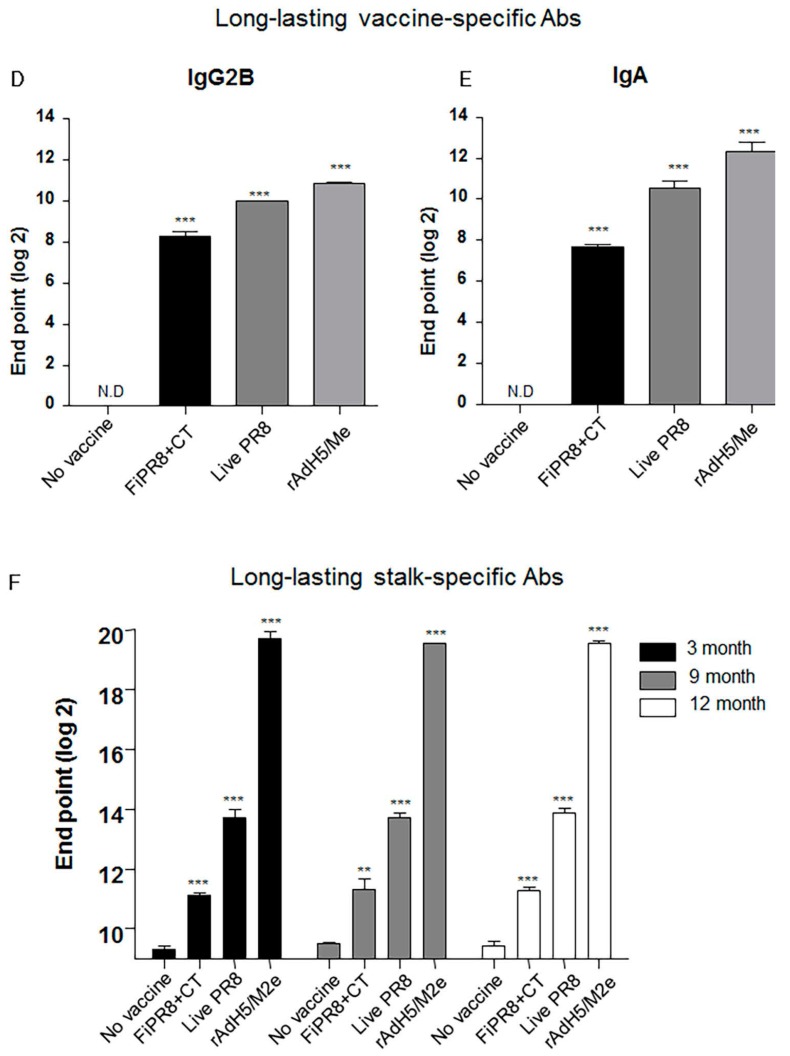
Intranasal immunization with adenovirus-vectored influenza vaccine induced long-lived HA stalk-specific humoral immunity. To detect long-term stalk-specific antibody responses, Balb/c mice were intranasally immunized with FiPR8+CT, live PR8, or rAdH5/M2e. Sera were collected from immunized mice at twelve moths post-immunization, and the levels of Th1/Th2 Abs were determined by a Mouse immunoglobulin Isotyping Magnetic Bead Panel assay (Millipore, Millipore Corporation, Billerica, MA, USA) (**B**–**E**). To examine the duration of the vaccine-specific and stalk-specific antibody responses, sera were collected from the immunized mice at three, nine, or twelve months post-immunization. The levels of vaccine-specific and stalk-specific Abs were determined by ELISA using plates coated with H1N1 (**A**), or baculorvirus-expressed cH9/1 protein (**F**). The values represent the mean ± SEM (vertical bars) end point ELISA antibody titers determined from five mice per group (*** *p* < 0.0001).

Since a desirable attribute of an effective influenza vaccines is the induction of durable vaccine responses that could provide protection over several influenza seasons, we next addressed whether the immunogenicity of the specific vaccines translated into long-lasting antibody responses. We examined the duration of the vaccine-induced humoral immunity for one year. As shown in Figure 5A, examination of antibody titers at three, nine, and 12 months post-immunization of mice with rAdH5/M2e, live PR8 or FiPR8+CT generated significant levels of long-lasting vaccine-specific Ab titers. Consistent with our previous IgG and IgA data (Figure 2), mice immunized with rAdH5/M2e, live PR8 or FiPR8+CT induced durable vaccine-specific IgG1, IgG2A, IgG2B, IgA titers (Figure 5B–E).

Next, we assessed the duration of the hemagglutinin stalk-specific antibody responses induced by each vaccination strategy. As shown in Figure 5F, and consistent with our findings presented in Figure 2, HA stalk-specific antibodies could be detected for all vaccination groups at 12 months after immunizations. Most notably, immunization with rAdH5/M2e significantly induced persistent levels of HA stalk-specific Abs that surpassed those induced by infection with wild-type influenza virus (Figure 5F). To assess the quality of protection provided by these durable antibody responses, we conducted passive immunization experiments in which naïve recipient mice received convalescent immune sera prepared at 12 months post-immunization. The passively immunized naïve recipient mice were then challenged by homosubtypic or heterosubtypic challenge infection. The convalescent sera from rAdH5/M2e-immunized mice protected the naïve recipient mice against homotypic and heterosubtypic challenge infections (data not shown). Consistent with the morbidity and mortality shown in Figure 4C,D, convalescent sera transferred from mice immunized with FiPR8+CT protected naïve recipient mice from homotypic H1N1 virus infection, but not from heterosubtypic H5N2 virus infection (data not shown). These results emphasize that long-term immunization with a vectored influenza virus vaccine, but not inactivated influenza virus vaccine, can induce prolonged Ab-dependent cross-protection against influenza viruses.

### 3.6. Formaldehyde Treatment Reduced Binding of HA Stalk-Specific Antibodies

**Figure 6:**
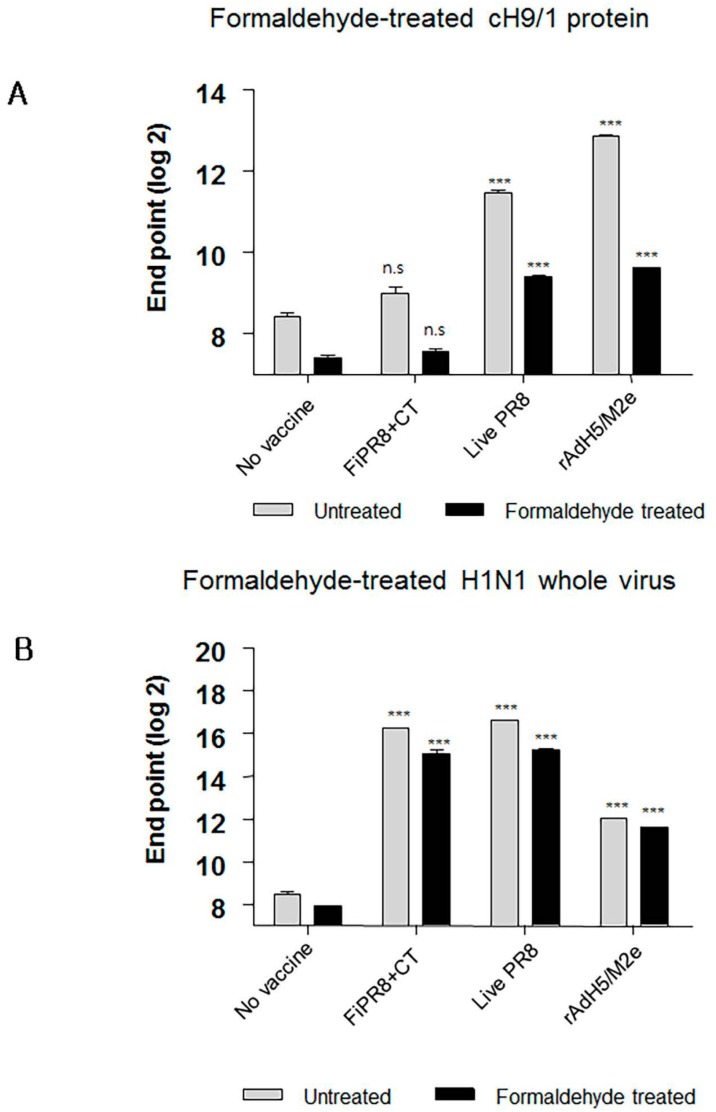
Formaldehyde treatment specifically reduces antigenicity of the HA stalk. To examine whether formaldehyde treatment alters the antigenicity of the HA, immune sera were examined by ELISA with formaldehyde-treated or untreated baculorvirus-expressed cH9/1 protein (**A**) or with formaldehyde-treated or untreated H1N1 whole virus particles (**B**). The values represent the mean ± SEM (vertical bars) end point ELISA antibody titers determined from five mice per group. The data represent results from three independent experiments (*** *p* < 0.0001).

**Figure 7:**
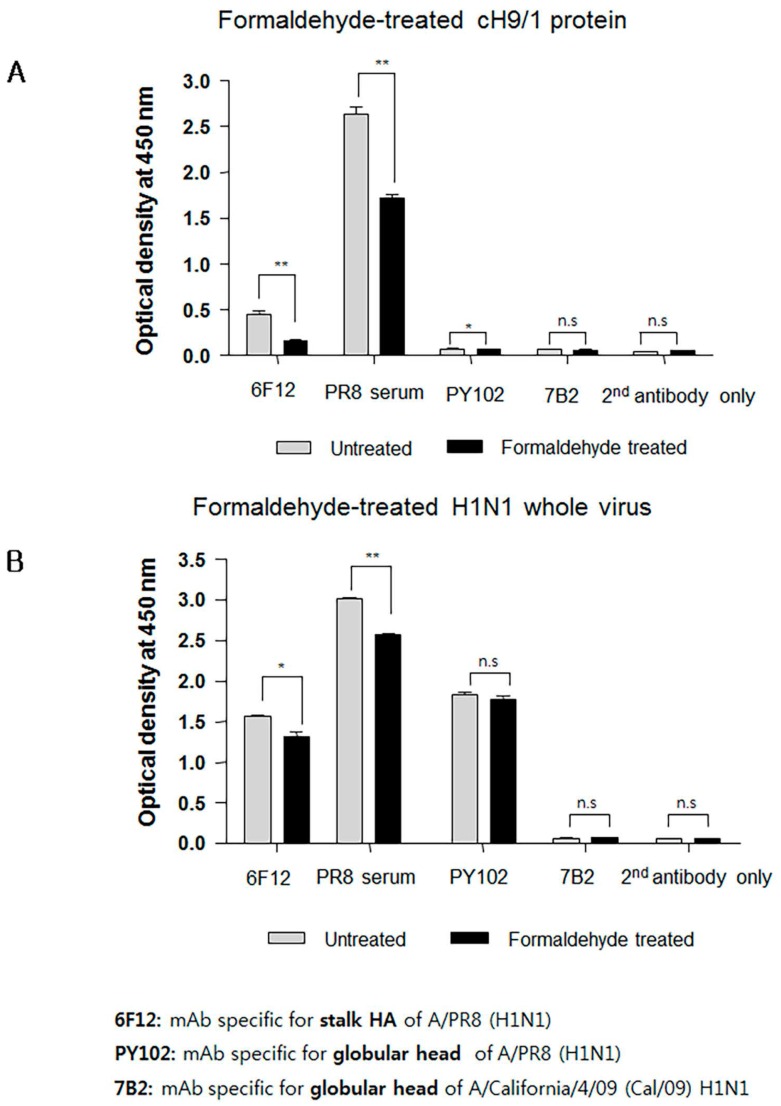
Formaldehyde treatment reduces the binding of a conformation-specific monoclonal antibody (mAb) to the HA stalk. ELISA were performed using mAb 6F12, mAb PY102, and mAb 7B2 that are specific for the PR8 HA stalk, globular head of PR8 virus, and globular head of A/California/4/09 (Cal/09) H1N1 virus, respectively. ELISA plates were coated with formaldehyde-treated or untreated chimeric protein (cH9/1) (**A**) or with formaldehyde-treated or untreated whole PR8 virus particles (**B**). Secondary (2nd) horseradish peroxidase (HRP)-conjugated Ab alone was included as the assay control. The data represent results from three independent ELISA with six wells per antigen in 96-well plates (* *p* < 0.01, ** *p* < 0.005).

Interestingly, sera from mice immunized with FiPR8+CT failed to provide cross-protection in the passive immunization experiments, and lacked high levels of stalk-specific Abs. We therefore examined by ELISA that included the cH9/1 hemagglutinin whether formaldehyde treatment could change the immunogenicity and/or antigenicity of the HA stalk domain, which, in terms of inactivated vaccines, could result in the induction of Abs that fail to recognize the native form of hemagglutinin produced during virus infection. As shown in Figure 6A, formaldehyde treatment significantly impacted the antigenicity of the HA stalk domain as evidenced by the reduced binding of Abs present in immune sera from immunized mice (Figure 6A). In contrast, treatment of whole PR8 virus with formaldehyde reduced the binding of Abs present in the immune sera to virus particles (Figure 6B). These results suggested that the antigenicity of the HA stalk domain is susceptible to formaldehyde treatment. To more specifically address this observation, we employed monoclonal antibodies (mAb) 6F12 that is specific for the HA stalk of A/PR/8/34 (PR8) (H1N1), PY102 that is specific for the globular head of A/PR/8/34 (PR8) (H1N1), and 7B2 that is specific for the globular head of A/California 4/09 (Cal09) (H1N1) in the binding assays. Plates coated with recombinant cH9/1 or whole PR8 virus particles were used in ELISA to determine whether or not formaldehyde treatment of the antigens impacted their antigenicity as evidenced by reduced binding by the mAbs. As shown in Figure 7A, formaldehyde treatment reduced remarkably binding of the stalk domain by the stalk-specific mAb (6F12) as well as by convalescent serum raised by infection of mice with a sublethal dose of wild type influenza virus. In contrast, treatment of whole virus particles with formaldehyde did not alter significantly the binding of the virus by head-specific mAb PY102 (Figure 7B). These results indicated that formaldehyde treatment substantially altered the antigenicity of the conserved stalk domain but had a lesser effect on the HA globular head.

### 3.7. Formaldehyde Treatment Reduced Monoclonal Antibody Binding to the Conserved Proteins NP and M2

**Figure 8:**
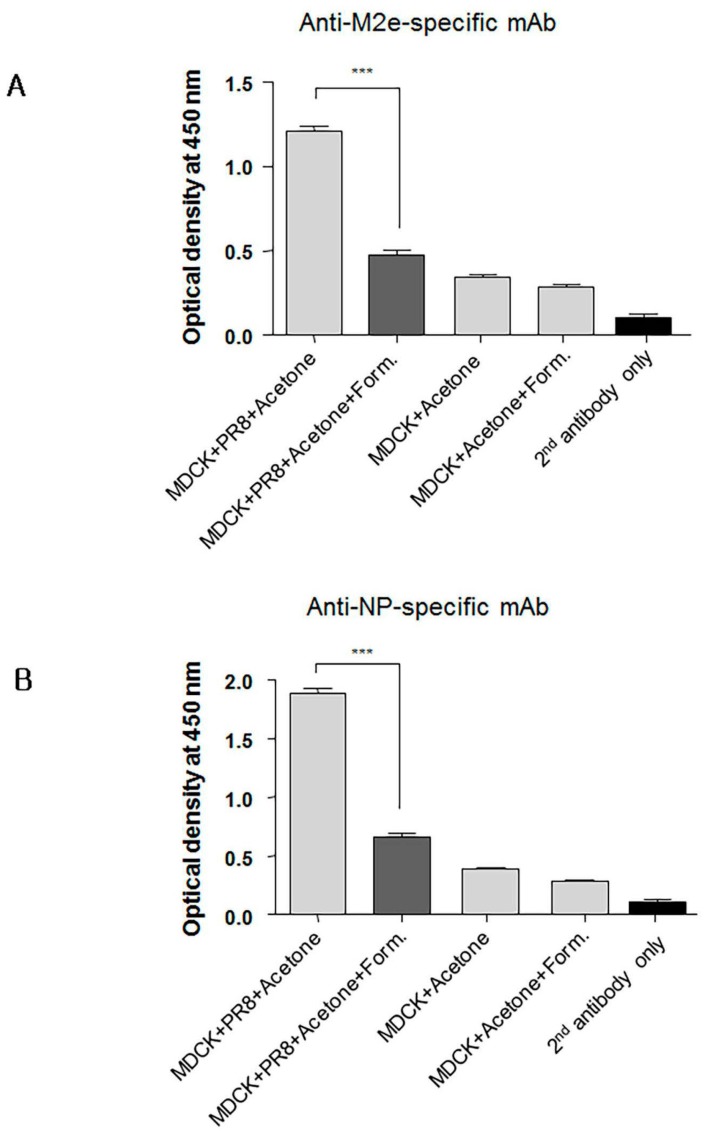
Formaldehyde treatment alters in the antigenicity of the M2 and NP viral proteins. Confluent MDCK cells cultured in 96-well flat bottom plates were infected with influenza A virus (IAV) to provide expression of nucleoprotein (NP) and M2. The plates were then treated with acetone and subsequently with formaldehyde or PBS for use in cell-based ELISA for detection of M2e-(**A**), or NP-specific monoclonal antibodies (mAbs) (**B**). Non-infected MDCK cells were included to determine level of background. Secondary (2nd) HRP-conjugated antibody alone was used as a negative control. The data represent results from three independent ELISA with six wells per antigen in 96-well plates (*** *p* < 0.0001).

Since immunity against conserved viral proteins can contribute to cross-protection, we next asked whether formaldehyde treatment of conserved proteins such as nucleoprotein (NP) and M2 also reduced their binding by specific monoclonal antibodies (mAbs). Cultured MDCK cells were infected overnight with PR8 virus to allow expression of M2 and NP. The infected cells were then treated with 80% acetone in phosphate buffered saline (PBS) and subsequently with 0.2% formaldehyde for use in ELISA. Cell-based ELISA was then performed using anti-M2 ectodomain (M2e) or anti-NP specific mAbs. As expected, formaldehyde treatment of the infected MDCK cells reduced significantly binding of NP- and M2e-specific mAbs (Figure 8A,B) indicating that formaldehyde treatment similarly impacted the antigenicity of the M2e and NP. Non-infected MDCK cells treated with 0.2% of formaldehyde were included to determine background staining. These results collectively emphasize the importance of preserving the native forms of conserved viral antigens such as M2e, NP, and the HA stalk domain for induction of Abs that mediate cross-protection. In contrast to inactivated vaccines, live vaccines, such as our adenovirus vectored influenza vaccine, are preferable because of induction of broadly cross-reactive Abs and presentation of viral proteins in their native conformation to the host’s immune system.

## 4. Discussion

It is generally accepted that live, attenuated influenza vaccines are superior to inactivated or subunit vaccines in terms of quality of the antiviral immune response, and the level and duration of protective immunity [48]. Numerous studies have reported on the importance of mucosal IgA responses in protection against influenza virus infection and disease [49,50,51,52]. The role of intranasal immunization and induction of IgA in protection against influenza virus replication in lungs or protection from disease are well established [53,54,55,56]. Recombinant adenovirus vectored vaccines are an attractive immunization approach given these vectors can express the antigen of interest and produce pathogen associated molecular patterns (PAMPS) that stimulate protective immune responses in the respiratory tract.

Here we have shown that immunization of mice with formaldehyde inactivated influenza virus vaccine, live influenza virus, or rAdH5/M2e elicited equivalent levels of hemagglutination inhibiting (HI) Abs neutralizing Abs (Figure 1A,B), and vaccine-specific IgG and IgA responses (Figure 1C–H).

In experimental animal model systems, the prophylactic and therapeutic potential of broadly neutralizing antibodies against the conserved influenza virus HA stalk domain have recently been evaluated with encouraging results [28,36,41,44,57,58,59,60,61]. Vaccine design approaches that target the conserved HA stalk can be informed by data obtained from studies on these monoclonal antibodies and are further supported by promising results generated from preclinical animal model studies [7,27,32,33]. As shown in Figure 1C–G, immunization with our adenovirus-vectored vaccine induced significant levels of vaccine-specific IgG, IgG1, IgG2A, and IgG2B Abs, as well as induced remarkable levels of HA stalk-specific Abs (Figure 2A–E). We found that immunization with rAdH5/M2e induced levels of stalk-specific IgG2A and IgG2B that were comparable to levels induced by live PR8 and superior to those induced by inactivated vaccines (Figure 2D,E).

The Th1/Th2 phenotype of the T cell response can profoundly influence B cell responses [29,34,50,52]. In the mouse model, IgG2A and IgG2B provide better protection against viral infections than IgG1 Abs [62,63]. The Th1/Th2 phenotype of the immune response induced by rAdH5/M2e immunization is critical for the type of immunoglobulins induced and level of protection of the respiratory tract. In this study, we observed that rAdH5/M2e immunization induced secretion of Th2 type cytokines, IL-4, IL-6, and IL-10 (Figure 3). These Th2 cytokines are important for proliferation and differentiation of B cells, isotype class switching, regulation in humoral immunity [11,13], and accelerated inflammatory responses [47]. The elevated levels of Th2-type cytokines are consistent with superior levels of HA stalk-specific IgG1 and IgA induced by rAdH5/M2e immunization. Cholera toxin is a strong agonist of IL-4 responses [46] and accordingly we noted that immunization with FiPR8+CT induced the highest levels of IL-4. Although we observed that immunization with rAdH5/M2e resulted in a lower level of IL-4 production as compared to immunization with FiPR8+CT, these responses further indicate that rAdH5/M2e immunization induces a Th2 response. Interestingly, these Th2 responses are not sufficient to completely counteract Th1 responses as indicated by the levels of HA-stalk specific IgG2A and IgG2B induced by rAdH5/M2e immunization. Interestingly, the elevated levels of IL-10 induced by immunization with live PR8 and rAdH5/M2e suggests the possible activation of regulatory T cells (Treg) during the adaptive immune response.

Passive immunization experiments demonstrated that sera from mice immunized with the vectored vaccine provided cross-protection against infection with different influenza virus subtypes; however, sera from mice immunized with formaldehyde-inactivated influenza virus failed to provide protection against heterosubtypic challenge (Figure 4A–D). Importantly, immunization with the rAdH5/M2e vaccine induced prolonged vaccine-specific (Figure 5A–E) and stalk-specific Abs as compared to immunization with formaldehyde-inactivated influenza virus or live influenza virus (Figure 5F). These findings suggest that the balanced Th1/Th2 HA stalk-specific antibody responses, specifically IgG1, IgG2A, IgG2B, and IgA antibodies, provided cross-protection from morbidity and mortality resulting from heterosubtypic influenza virus infection.

Formaldehyde is one of the earliest and most widely used chemical methods to inactivate virus for vaccine production; however, this method of treatment can cause irreversible modifications by cross-linking antigens and damaging key antigenic epitopes leading to reduced immunogenicity [64,65]. It has been reported from studies that compared immune responses to live influenza virus against seasonal formaldehyde-inactivated influenza virus vaccine (i.e., TIV) that: (i) live influenza virus induced significantly better protection than inactivated vaccine in infants and young children; (ii) elicited stronger influenza virus-specific serum Ab levels; and (iii) stimulated greater T-cell responses that could provide cross-protection against heterologous challenge in small animal models of influenza virus disease [5,66,67]. We conjectured that the HA stalk domain and more importantly its native epitopes play an important role in development of protective immunity against infection with heterosubtypic influenza viruses. As shown in Figure 6A and Figure 7A, we found that formaldehyde treatment of baculovirus-expressed chimeric HA protein (cH9/1) reduced binding of HA stalk domain-specific Abs. However, formaldehyde treatment did not significantly reduce binding of Abs to virus particles (Figure 6B), or alter binding of HA head-specific mAbs to whole virus particles (Figure 7B). These findings suggest that formaldehyde treatment altered antigenic epitopes, and thus the antigenicities, of the HA stalk, the M2e ectodomain, and NP protein (Figure 8A,B), and support the speculation that recognition of the native conformation of the HA stalk domain is important for heterosubtypic immunity. Our results also indicate that immunization with rAdH5/M2e induced Abs that are specific for native HA stalk epitopes that contributed to cross-protection against other influenza virus subtypes (Figure 3D and Figure 4A). Thus, immunization with rAdH5/M2e induced long-lived antibody responses that recognize native epitopes of the HA stalk.

In summary, our study demonstrated that our adenovirus-vectored influenza vaccine, rAdH5/M2e, induces a balanced Th1/Th2 immune responses that provide durable cross-protection against infection with influenza viruses. Our results further indicate that preserving the native conformations of HA antigenic epitopes, such as accomplished by our adenovirus-vectored influenza vaccine, is important for induction of neutralizing antibodies, and in particular stalk-binding Abs that form the basis of broadly protective immunity against heterologous influenza virus challenge. Our findings are of relevance to the manufacture of seasonal influenza vaccines and will likely influence the development of novel universal influenza vaccines that are based on the conserved HA stalk domain.

## References

[1] Clover R.D., Crawford S., Glezen W.P., Taber L.H., Matson C.C., Couch R.B. (1991). Comparison of heterotypic protection against influenza A/Taiwan/86 (H1N1) by attenuated and inactivated vaccines to A/chile/83-like viruses. J. Infect. Dis..

[2] Chen G.L., Min J., Lamirande E.W., Santos C., Jin H., Kemble G., Subbarao K. (2011). Comparison of a live attenuated 2009 H1N1 vaccine with seasonal influenza vaccines against 2009 pandemic H1N1 virus infection in mice and ferrets. J. Infect. Dis..

[3] Cheng X., Zengel J.R., Suguitan A.L., Xu Q., Wang W., Lin J., Jin H. (2013). Evaluation of the humoral and cellular immune responses elicited by the live attenuated and inactivated influenza vaccines and their roles in heterologous protection in ferrets. J. Infect. Dis..

[4] Krammer F., Pica N., Hai R., Margine I., Palese P. (2013). Chimeric hemagglutinin influenza virus vaccine constructs elicit broadly protective stalk-specific antibodies. J. Virol..

[5] Chen Z., Santos C., Aspelund A., Gillim-Ross L., Jin H., Kemble G., Subbarao K. (2009). Evaluation of live attenuated influenza a virus H6 vaccines in mice and ferrets. J. Virol..

[6] Rao S.S., Kong W.P., Wei C.J., Van Hoeven N., Gorres J.P., Nason M., Andersen H., Tumpey T.M., Nabel G.J. (2010). Comparative efficacy of hemagglutinin, nucleoprotein, and matrix 2 protein gene-based vaccination against H5N1 influenza in mouse and ferret. PLoS ONE.

[7] Steel J., Lowen A.C., Wang T.T., Yondola M., Gao Q., Haye K., Garcia-Sastre A., Palese P. (2010). Influenza virus vaccine based on the conserved hemagglutinin stalk domain. mBio.

[8] Delavenne X., Zufferey P., Baylot D., Nguyen P., Borg J.Y., Fontenay M., Deygas B., Mismetti P., Laporte S. (2010). Population pharmacokinetics of fondaparinux administered at prophylactic doses after major orthopaedic surgery in everyday practice. Thromb. Haemost..

[9] Belshe R.B., Edwards K.M., Vesikari T., Black S.V., Walker R.E., Hultquist M., Kemble G., Connor E.M., CAIV-T Comparative Efficacy Study Group (2007). Live attenuated versus inactivated influenza vaccine in infants and young children. N. Engl. J. Med..

[10] Lee I.S., Liu Y., Narazaki M., Hibi M., Kishimoto T., Taga T. (1997). Vav is associated with signal transducing molecules gp130, Grb2 and Erk2, and is tyrosine phosphorylated in response to interleukin-6. FEBS Lett..

[11] Kingsley C.I., Karim M., Bushell A.R., Wood K.J. (2002). CD25+CD4+ regulatory T cells prevent graft rejection: CTLA-4- and IL-10-dependent immunoregulation of alloresponses. J. Immunol..

[12] Sui J., Hwang W.C., Perez S., Wei G., Aird D., Chen L.M., Santelli E., Stec B., Cadwell G., Ali M. (2009). Structural and functional bases for broad-spectrum neutralization of avian and human influenza a viruses. Nat. Struct. Mol. Biol..

[13] Yu X., Zhang X., Zhao B., Wang J., Zhu Z., Teng Z., Shao J., Shen J., Gao Y., Yuan Z. (2011). Intensive cytokine induction in pandemic H1N1 influenza virus infection accompanied by robust production of IL-10 and IL-6. PLoS ONE.

[14] Stillé C.J. (2005). Major-General Anthony Wayne and the Pennsylvania Line in the Continental Army.

[15] Antel J.P. (2005). Clinical Neuroimmunology.

[16] Holgate S.T. (2008). Are long-acting β2-agonists safe in the treatment of asthma?. Pol. Arch. Med. Wewn..

[17] Holgate S.T., Polosa R. (2008). Treatment strategies for allergy and asthma. Nat. Rev. Immunol..

[18] Jian Y.R., Chang S.Y., Lin P.Y., Yang Y.H., Chuang Y.H. (2013). Inactivated influenza virus vaccine is efficient and reduces IL-4 and IL-6 in allergic asthma mice. Influenza Other Respir. Viruses.

[19] Van Kampen K.R., Shi Z., Gao P., Zhang J., Foster K.W., Chen D.T., Marks D., Elmets C.A., Tang D.C. (2005). Safety and immunogenicity of adenovirus-vectored nasal and epicutaneous influenza vaccines in humans. Vaccine.

[20] Fiers W., De Filette M., El Bakkouri K., Schepens B., Roose K., Schotsaert M., Birkett A., Saelens X. (2009). M2e-based universal influenza a vaccine. Vaccine.

[21] Schotsaert M., De Filette M., Fiers W., Saelens X. (2009). Universal M2 ectodomain-based influenza a vaccines: Preclinical and clinical developments. Expert Rev. Vaccines.

[22] Ellebedy A.H., Krammer F., Li G.M., Miller M.S., Chiu C., Wrammert J., Chang C.Y., Davis C.W., McCausland M., Elbein R. (2014). Induction of broadly cross-reactive antibody responses to the influenza HA stem region following H5N1 vaccination in humans. Proc. Natl. Acad. Sci. USA.

[23] Epstein S.L., Kong W., Misplon J.A., Lo C.Y., Tumpey T.M., Xu L., Nabel G.J. (2005). Protection against multiple influenza a subtype by vaccination. Vaccine.

[24] Munder M., Eichmann K., Moran J.M., Centeno F., Soler G., Modolell M. (1999). Th1/Th2-regulated expression of arginase isoforms in murine macrophages and dendritic cells. J. Immunol..

[25] Tomy Joseph J.M., Lu B., Vogel L., Swayne D., Jin H., Kemble G., Subbarao K. (2008). A live attenuated cold-adapted influenza a H7N3 virus vaccine provides protection against homologous and heterologous H7 viruses in mice and ferrets. Virology.

[26] Quan F.S., Compans R.W., Nguyen H.H., Kang S.M. (2008). Induction of heterosubtypic immunity to influenza virus by intranasal immunization. J. Virol..

[27] Kim E.H., Park H.J., Han G.Y., Song M.K., Pereboev A., Hong J.S., Chang J., Byun Y.H., Seong B.L., Nguyen H.H. (2014). Intranasal adenovirus-vectored vaccine for induction of long-lasting humoral immunity-mediated broad protection against influenza in mice. J. Virol..

[28] Kolpe A., Schepens B., Fiers W., Saelens X. (2017). M2-based influenza vaccines: Recent advances and clinical potential. Expert Rev. Vaccines.

[29] Lee Y.N., Kim M.C., Lee Y.T., Kim Y.J., Kang S.M. (2015). Mechanisms of cross-protection by influenza virus M2-based vaccines. Immune Netw..

[30] Eliasson D.G., Omokanye A., Schon K., Wenzel U.A., Bernasconi V., Bemark M., Kolpe A., El Bakkouri K., Ysenbaert T., Deng L. (2017). M2e-tetramer-specific memory CD4 T cells are broadly protective against influenza infection. Mucosal Immunol..

[31] Kreijtz J.H., Suezer Y., de Mutsert G., van den Brand J.M., van Amerongen G., Schnierle B.S., Kuiken T., Fouchier R.A., Lower J., Osterhaus A.D. (2009). Recombinant modified vaccinia virus ankara expressing the hemagglutinin gene confers protection against homologous and heterologous H5N1 influenza virus infections in macaques. J. Infect. Dis..

[32] Bommakanti G., Citron M.P., Hepler R.W., Callahan C., Heidecker G.J., Najar T.A., Lu X., Joyce J.G., Shiver J.W., Casimiro D.R. (2010). Design of an HA2-based escherichia coli expressed influenza immunogen that protects mice from pathogenic challenge. Proc. Natl. Acad. Sci. USA.

[33] Krammer F. (2015). The quest for a universal flu vaccine: Headless Ha 2.0. Cell Host Microbe.

[34] Stevens T.L., Bossie A., Sanders V.M., Fernandez-Botran R., Coffman R.L., Mosmann T.R., Vitetta E.S. (1988). Regulation of antibody isotype secretion by subsets of antigen-specific helper T cells. Nature.

[35] Moran T.M., Park H., Fernandez-Sesma A., Schulman J.L. (1999). Th2 responses to inactivated influenza virus can be converted to Th1 responses and facilitate recovery from heterosubtypic virus infection. J. Infect. Dis..

[36] Mosmann T.R., Coffman R.L. (1989). Heterogeneity of cytokine secretion patterns and functions of helper T cells. Adv. Immunol..

[37] Coffman R.L., Lebman D.A., Rothman P. (1993). Mechanism and regulation of immunoglobulin isotype switching. Adv. Immunol..

[38] Spellberg B., Edwards J.E. (2001). Type 1/type 2 immunity in infectious diseases. Clin. Infect. Dis..

[39] Diehl S., Chow C.W., Weiss L., Palmetshofer A., Twardzik T., Rounds L., Serfling E., Davis R.J., Anguita J., Rincon M. (2002). Induction of NFATc2 expression by interleukin 6 promotes T helper type 2 differentiation. J. Exp. Med..

[40] Mogensen T.H., Paludan S.R. (2001). Molecular pathways in virus-induced cytokine production. Microbiol. Mol. Biol. Rev..

[41] Wrammert J., Koutsonanos D., Li G.M., Edupuganti S., Sui J., Morrissey M., McCausland M., Skountzou I., Hornig M., Lipkin W.I. (2011). Broadly cross-reactive antibodies dominate the human B cell response against 2009 pandemic H1N1 influenza virus infection. J. Exp. Med..

[42] Nguyen H.H., Tumpey T.M., Park H.J., Byun Y.H., Tran L.D., Nguyen V.D., Kilgore P.E., Czerkinsky C., Katz J.M., Seong B.L. (2010). Prophylactic and therapeutic efficacy of avian antibodies against influenza virus H5N1 and H1N1 in mice. PLoS ONE.

[43] Shim B.S., Choi Y.K., Yun C.H., Lee E.G., Jeon Y.S., Park S.M., Cheon I.S., Joo D.H., Cho C.H., Song M.S. (2011). Sublingual immunization with M2-based vaccine induces broad protective immunity against influenza. PLoS ONE.

[44] Hai R., Krammer F., Tan G.S., Pica N., Eggink D., Maamary J., Margine I., Albrecht R.A., Palese P. (2012). Influenza viruses expressing chimeric hemagglutinins: Globular head and stalk domains derived from different subtypes. J. Virol..

[45] Tan G.S., Krammer F., Eggink D., Kongchanagul A., Moran T.M., Palese P. (2012). A pan-H1 anti-hemagglutinin monoclonal antibody with potent broad-spectrum efficacy in vivo. J. Virol..

[46] Xu-Amano J., Jackson R.J., Fujihashi K., Kiyono H., Staats H.F., McGhee J.R. (1994). Helper Th1 and Th2 cell responses following mucosal or systemic immunization with cholera toxin. Vaccine.

[47] Taga T., Kishimoto T. (1997). Gp130 and the interleukin-6 family of cytokines. Annu. Rev. Immunol..

[48] Amanna I.J., Slifka M.K. (2009). Wanted, dead or alive: New viral vaccines. Antivir. Res..

[49] Arulanandam B.P., Raeder R.H., Nedrud J.G., Bucher D.J., Le J., Metzger D.W. (2001). Iga immunodeficiency leads to inadequate Th cell priming and increased susceptibility to influenza virus infection. J. Immunol..

[50] Van Riet E., Ainai A., Suzuki T., Hasegawa H. (2012). Mucosal IgA responses in influenza virus infections; thoughts for vaccine design. Vaccine.

[51] Tamura S., Kurata T. (2004). Defense mechanisms against influenza virus infection in the respiratory tract mucosa. Jpn. J. Infect. Dis..

[52] Gorse G.J., Otto E.E., Powers D.C., Chambers G.W., Eickhoff C.S., Newman F.K. (1996). Induction of mucosal antibodies by live attenuated and inactivated influenza virus vaccines in the chronically ill elderly. J. Infect. Dis..

[53] Brandtzaeg P. (2003). Role of mucosal immunity in influenza. Dev. Biol..

[54] Haan L., Verweij W.R., Holtrop M., Brands R., van Scharrenburg G.J., Palache A.M., Agsteribbe E., Wilschut J. (2001). Nasal or intramuscular immunization of mice with influenza subunit antigen and the B subunit of escherichia coli heat-labile toxin induces IgA- or IgG-mediated protective mucosal immunity. Vaccine.

[55] Plante M., Jones T., Allard F., Torossian K., Gauthier J., St-Felix N., White G.L., Lowell G.H., Burt D.S. (2001). Nasal immunization with subunit proteosome influenza vaccines induces serum HAI, mucosal IgA and protection against influenza challenge. Vaccine.

[56] Verweij W.R., de Haan L., Holtrop M., Agsteribbe E., Brands R., van Scharrenburg G.J., Wilschut J. (1998). Mucosal immunoadjuvant activity of recombinant escherichia coli heat-labile enterotoxin and its B subunit: Induction of systemic IgG and secretory IgA responses in mice by intranasal immunization with influenza virus surface antigen. Vaccine.

[57] Dreyfus C., Laursen N.S., Kwaks T., Zuijdgeest D., Khayat R., Ekiert D.C., Lee J.H., Metlagel Z., Bujny M.V., Jongeneelen M. (2012). Highly conserved protective epitopes on influenza B viruses. Science.

[58] Ekiert D.C., Bhabha G., Elsliger M.A., Friesen R.H., Jongeneelen M., Throsby M., Goudsmit J., Wilson I.A. (2009). Antibody recognition of a highly conserved influenza virus epitope. Science.

[59] Suguitan A.L., McAuliffe J., Mills K.L., Jin H., Duke G., Lu B., Luke C.J., Murphy B., Swayne D.E., Kemble G. (2006). Live, attenuated influenza a H5N1 candidate vaccines provide broad cross-protection in mice and ferrets. PLoS Med..

[60] Thomson C.A., Wang Y., Jackson L.M., Olson M., Wang W., Liavonchanka A., Keleta L., Silva V., Diederich S., Jones R.B. (2012). Pandemic H1N1 influenza infection and vaccination in humans induces cross-protective antibodies that target the hemagglutinin stem. Front. Immunol..

[61] Throsby M., van den Brink E., Jongeneelen M., Poon L.L., Alard P., Cornelissen L., Bakker A., Cox F., van Deventer E., Guan Y. (2008). Heterosubtypic neutralizing monoclonal antibodies cross-protective against H5N1 and H1N1 recovered from human IgM+ memory B cells. PLoS ONE.

[62] Coucke D., Schotsaert M., Libert C., Pringels E., Vervaet C., Foreman P., Saelens X., Remon J.P. (2009). Spray-dried powders of starch and crosslinked poly(acrylic acid) as carriers for nasal delivery of inactivated influenza vaccine. Vaccine.

[63] Markine-Goriaynoff D., Coutelier J.P. (2002). Increased efficacy of the immunoglobulin G2a subclass in antibody-mediated protection against lactate dehydrogenase-elevating virus-induced polioencephalomyelitis revealed with switch mutants. J. Virol..

[64] Brown F. (1993). Review of accidents caused by incomplete inactivation of viruses. Dev. Biol. Stand..

[65] Branch A. (1938). Vaccination against whooping cough. Can. Med. Assoc. J..

[66] Hoft D.F., Babusis E., Worku S., Spencer C.T., Lottenbach K., Truscott S.M., Abate G., Sakala I.G., Edwards K.M., Creech C.B. (2011). Live and inactivated influenza vaccines induce similar humoral responses, but only live vaccines induce diverse T-cell responses in young children. J. Infect. Dis..

[67] Houser K.V., Katz J.M., Tumpey T.M. (2013). Seasonal trivalent inactivated influenza vaccine does not protect against newly emerging variants of influenza a (H3N2v) virus in ferrets. J. Virol..

